# Cognitive functioning and predictors thereof in patients with 1–10 brain metastases selected for stereotactic radiosurgery

**DOI:** 10.1007/s11060-019-03292-y

**Published:** 2019-09-24

**Authors:** Wietske C. M. Schimmel, Karin Gehring, Patrick E. J. Hanssens, Margriet M. Sitskoorn

**Affiliations:** 1 Gamma Knife Center, Elisabeth-TweeSteden Hospital, Tilburg, The Netherlands; 2 Department of Neurosurgery, Elisabeth-TweeSteden Hospital, Tilburg, The Netherlands; 3 Department of Cognitive Neuropsychology, Tilburg University, Tilburg, The Netherlands; 4 Department of Cognitive Neuropsychology, Tilburg University, Simon Building; Room S221, P.O. Box 90153, 5000 LE Tilburg, The Netherlands

**Keywords:** Brain metastases, Cognitive functioning, Stereotactic radiosurgery, Gamma knife radiosurgery

## Abstract

**Purpose:**

Information on predictive factors of cognitive functioning in patients with (multiple) brain metastases (BM) selected for radiosurgery may allow for more individual care and may play a role in predicting cognitive outcome after radiosurgery. The aim of this study was to evaluate cognitive performance, and predictors thereof, in patients with 1–10 BM before radiosurgery.

**Methods:**

Cognition was measured before radiosurgery using a standardized neuropsychological test battery in patients with 1–10 BM (expected survival > 3 months; KPS ≥ 70; no prior BM treatment). Regression formulae were constructed to calculate sociodemographically corrected z scores. Group and individual cognitive functioning was analyzed. Multivariable regression was used to explore potential predictors.

**Results:**

Patients (N = 92) performed significantly worse than controls (N = 104) on all 11 test variables (medium-large effect sizes for 8 variables). Percentages of impairment were highest for information processing (55.3%), dexterity (43.2%) and cognitive flexibility (28.7%). 62% and 46% of patients had impairments in at least two, or three test variables, respectively. Models including combinations of clinical and psychological variables were predictive of verbal memory, psychomotor speed, information processing and dexterity. Neither number nor volume of metastases predicted patients’ test performance.

**Conclusions:**

Already before radiosurgery, almost half of the patients suffered from severe cognitive deficits in at least three test variables. At group and individual level, information processing, cognitive flexibility, and dexterity were most affected. These cognitive impairments may impair daily functioning and patients’ ability to make (shared) treatment decisions. Both clinical (symptomatic BM; timing of BM diagnosis) and psychological (mental fatigue) characteristics influenced cognitive performance.

**Clinical trial information:**

Cognition and Radiation Study A (CAR-Study A; ClinicalTrials.gov Identifier: NCT02953756; Medical Ethics Committee file number: NL53472.028.15/P1515).

**Electronic supplementary material:**

The online version of this article (10.1007/s11060-019-03292-y) contains supplementary material, which is available to authorized users.

## Introduction

Stereotactic radiosurgery (SRS) is increasingly applied in patients with brain metastases (BM) as it is expected to cause less cognitive damage than whole brain radiation therapy (WBRT) because it allows precise radiation delivery to the BM only. Patients with newly diagnosed BM who are accepted for SRS alone represent a selective group of patients with a relatively good performance status (Karnofsky Performance Status ≥ 70) and an expected survival time of at least three months [1]. Nonetheless, before BM treatment, many patients experience cognitive impairments that may be caused by several factors, including the BM itself, medication use, the primary cancer, or side effects of systemic treatment [2]. Thorough assessment and understanding of these impairments is of high relevance because these impairments, e.g., slow processing of information, may negatively affect patients’ ability to reason through (shared) medical treatment decisions, daily functioning and ultimately patients’ quality of life [3]. In addition, pretreatment neuropsychological assessment is crucial for the evaluation of cognitive changes after SRS [4].

There have been relatively few studies in patients with newly diagnosed BM who undergo SRS that evaluated (baseline) cognitive functions with objective neuropsychological tests, as opposed to insensitive measures for this purpose such as the Mini-Mental Status Examination (MMSE) [5]. Moreover, in reports thereof, baseline test results were not the primary focus and were only (very) briefly discussed. The majority of patients (ranging from 53 to 67%) in these studies showed mild to severe impairments in at least one cognitive domain. Executive function, verbal learning and memory, dexterity, information processing, and visuoconstruction were the cognitive domains most frequently affected [6–10], which is in line with research in patients with BM in general [11–15]. Previous studies, however, concerned patients with a limited number of BM 1–4 whereas the use of SRS is expanding to patients with multiple (> 4) BM [16–18]. More recently, total volume of BM, as opposed to their number, has gained interest as a predictor for outcomes of patients with BM (including overall survival, local control and distant progression of BM) [19–22]. However, thus far, only a few studies have examined the relationship between number and volume of BM and (pretreatment) cognitive functioning in patients with BM. In univariate analyses, a larger total volume of BM was suggested to be associated with worse baseline cognitive performance in four studies, including two small pilot studies [6, 8, 10, 15]. The number of BM was however not associated with cognitive performance in these studies, suggesting that cognitive functions are more affected by the total burden of BM than by the number of lesions [15]. To our knowledge only one previous study explored potential predictors of pretreatment cognition in patients with BM in a multivariable manner [15]. This study showed that total volume of BM was a predictor for baseline cognitive impairment in patients that were randomly assigned to WBRT with or without motexafin gadolinium.

In the current study, we investigated the incidence and severity of cognitive impairments in patients with 1 to 10 BM before Gamma Knife radiosurgery (GKRS). Both number and volume of BM are examined as potential predictors of baseline cognitive functioning. In addition, the role of other clinical variables (including KPS and diagnosis-specific graded prognostic assessment (DS-GPA [23]) and psychological variables, such as fatigue and symptoms of anxiety and depression, known to impact cognitive test performance [24–26], were explored.

## Methods and materials

Baseline test data of patients from the ongoing prospective longitudinal observational Cognition and Radiation Study A (CAR-Study A; ClinicalTrials.gov Identifier: NCT02953756) were analyzed. In addition, non-cancer controls were recruited. This study was approved by the Medical Ethics Committee Brabant (file NL53472.028.15/P1515).

### Patients

Adult patients were recruited at the Elisabeth-TweeSteden Hospital (ETZ; Tilburg, the Netherlands). Eligibility criteria were previously described by Verhaak et al. [27]. Most important inclusion criteria included: 1–10 newly diagnosed BM on a diagnostic or referral MRI-scan from a histologically proven malignant cancer, KPS ≥ 70, total tumor volume ≤ 30 cm^3^, and expected survival > 3 months. Exclusion criteria included: active primary brain tumor, small cell lung cancer, leptomeningeal metastases, or progressive symptomatic systemic disease without further treatment options, prior treatment directed at the BM (e.g., radiation therapy or surgery). Patients were screened by the radiation-oncologist during the first consultation. Neuropsychological assessment (NPA) was performed by a trained neuropsychologist in the morning before treatment.

### Non-cancer controls

A normative group of adult non-cancer controls, as previously described by Verhaak et al. [27], were recruited by convenience sampling from the general community and were selected to be, as much as possible, comparable to the general population and our patient-group, except for the fact that they were not allowed to have (a history of) cancer or severe cerebrovascular disease in the past year. Eligible controls received a study information letter and a medical checklist. All patients and controls signed informed consent before the NPA.

### Measures

Medical records were consulted to extract patient characteristics. BM diagnosed > 30 days from the diagnosis of the primary tumor were considered metachronous (all other BM were considered synchronous). A well-established test battery [2, 28] was used that consisted of six neuropsychological tests, generating 11 test variables. In addition, three questionnaires [29–31] were administered (Table 1). FACT-Br data was not evaluated in this study.

**Table 1:** Neuropsychological test battery including questionnaires

Neuropsychological test	Description/cognitive domain
Hopkins verbal learning test-revised (HVLT-R)	Verbal memory test (12 target words, 6 parallel versions)
1. HVLT-R immediate recall	Short-term verbal memory span
2. HVLT-R delayed recall	Longer-term verbal memory
3. HVLT-R recognition	Delayed verbal recognition (correct responses minus semantically related and unrelated false-positive errors)
Trail making test (TMT)	Test of visual conceptual and visuomotor tracking
4. TMT A	Psychomotor speed
5. TMT B	Cognitive flexibility (aspect of executive functioning)
Controlled oral word association test 6. COWA	Speeded verbal fluency test (requires aspects of executive functioning; 2 parallel versions)
WAIS digit span	Forward and backward repetitions of series of digits
7. Digit span forward	Immediate attention
8. Digit span backward	Working memory
WAIS digit symbol 9. Digit symbol	Symbol substitution test of information processing speed (requires visuomotor coordination and sustained attention)
Lafayette grooved pegboard (GP)	A manipulative dexterity test
10. GP dominant hand	Motor dexterity dominant hand
11. GP non-dominant hand	Motor dexterity non-dominant hand
Questionnaire	Description
Hospital and Anxiety and Depression Scale (HADS)	Symptoms of anxiety and depression
Multidimensional Fatigue Inventory (MFI)	Symptoms of general fatigue, physical fatigue, reduced activation, reduced motivation and mental fatigue
Functional assessment of cancer therapy-brain (FACT-Br)	General quality of life (QOL) questionnaire that reflects symptoms or problems associated with brain malignancies across five scales

*WAIS* Wechsler Adult Intelligence Scale

### Statistical analyses

Descriptive and comparative (Chi-square test; independent samples t-test) analyses were performed with respect to characteristics of patients and controls.

By means of multiple linear regression analyses, that regressed raw cognitive test scores of the control sample on age, sex and educational level, normative formulae were generated [32]. Raw Trails B scores were adjusted for sex, age, educational level and the Trails A score to derive the interference index. Sociodemographically-adjusted z scores were derived: Patients’ z score = patient’s raw score minus the predicted score divided by the SD of the control sample’s residuals. Higher z scores reflect better cognitive performance.

To compare cognitive performance between patients and controls, one-tailed one-sample z tests were performed. Patients’ mean z scores are equal to Glass’ delta effect sizes (Mean_Patients_ − Mean_Controls_ / SD_Controls_; [33]), where 0.2 = small, 0.5 = medium, and 0.8 = large effect [34]. Impaired cognitive performance was defined as a z-score ≤  − 1.5. Percentages of patients with impaired performance per test variable, and on one, two or more tests were calculated.

Correlations were explored of patients’ cognitive performances with clinical and psychological characteristics. A maximum of three additional predictors with the highest significant (*p* < 0.05) correlations were selected per test variable. Hierarchical multiple regression analyses were then performed to regress patients’ z scores on the selected predictors. In all models, number (dummy-coded) and volume of BM were entered separately in Block 1. To reduce false discovery rate (FDR) due to multiple testing, alpha’s were corrected per hypothesis, according to the Benjamini–Hochberg method [35]. All statistical analyses were performed with SPSS Statistics 25.0.

## Results

### Participants’ characteristics

In total, 92 patients and 104 controls were included. Patients and controls did not differ in terms of sex, age and education (Table 2). Forty percent of patients had more than three BM and the most common primary tumor was non-small cell lung cancer (NSCLC; 60%). Median total volume of BM was 5.64 cm^3^. For 16 patients (17.4%) and 5 controls (4.8%) scores on one or more tests were missing due to: invalid assessment (HVLT-R recognition, TMT), unfamiliarity with the alphabet (TMT), visual problems (TMT, Digit Symbol, GP), and impairments in dexterity (TMT, Digit Symbol, GP).

**Table 2:** Characteristics of patients and controls

	No. of patients (%)	No. of controls (%)	Test statistic	*p* value
Number of participants	92	104		
Sex			*χ*^2^ = 0.18^A^	0.67
Male	47 (51)	50 (48)		
Female	45 (49)	54 (52)		
Age in years, mean ± SD (range)	62 ± 10 (31–80)	59 ± 11 (31–87)	*t* = 1.53^B^	0.13
Educational level			*χ*^2^ = 4.63^A^	0.10
Low	28 (31)	25 (24)		
Middle	37 (40)	33 (32)		
High	27 (29)	46 (44)		
KPS				
70–80	33 (36)			
90–100	59 (64)	N/A		
DS-GPA				
Class I (3.5–4 points)	8 (9)	N/A		
Class II (2.5–3 points)	33 (35)			
Class III (1.5–2 points)	44 (48)			
Class IV (0–1 points)	7 (8)			
Primary cancer				
Lung (NSCLC)	55 (60)	N/A		
Renal	15 (16)			
Melanoma	12 (13)			
Other	10 (11)			
Number of BM				
1	32 (35)			
2–4	29 (31)	N/A		
5–10	31 (34)			
BM volume by patient (cm^3^)				
Median (range)	5.64 (0.02–31.15)	N/A		
Timing of BM diagnosis				
Synchronous	28 (30)			
Metachronous	64 (70)			
Extracranial metastases^a^				
Yes	66 (72)			
No	26 (28)	N/A		
BM Symptoms at diagnosis				
Symptomatic	64 (70)			
Asymptomatic	28 (30)	N/A		
Systemic therapy				
No	39 (42)	N/A		
Yes	53 (58)			
Chemotherapy^b^	37 (40)			
HADS scores^c^, mean ± SD				
Anxiety subscale	7.3 ± 4.4	4.4 ± 2.8	t = 5.36^B^	< 0.001
Depression subscale	5.7 ± 4.1	3.5 ± 2.9	t = 4.37^B^	< 0.001

Educational level according to Verhage (1964; 7 classes): low = 1–4, middle = 5, high = 6–7

*N/A* not applicable, *KPS* Karnofsky performance scale, *DS-GPA* diagnosis-specific graded prognostic assessment, *NSCLC* non-small cell lung cancer, *BM* brain metastases

^a^Including lymphatic metastases at baseline or before

^b^Alone or in combination with other systemic therapies

^c^Hospital Anxiety and Depression Scale with two 7-item subscales; range 0–21 points; higher scores indicate more symptoms of anxiety or depression

^A^Chi-square test of homogeneity

^B^Independent-samples T test

### Group-level cognitive performance

Patients performed significantly worse than non-cancer controls on all 11 test variables with medium to large effect sizes for 8 out of 11 variables (Table 3). Lowest performance was found on measures of psychomotor speed, cognitive flexibility, information processing, and dexterity of both dominant and non-dominant hand.

**Table 3:** Cognitive performance at group and individual level

Test variables	Group level				Individual level			
	Mean Z scores of patients versus controls^a^				Impaired performance per test variable^b^			
	z score d	z test	*p* value	Effect size^e^	Patients (%)	Controls (%)	*χ*^*2*^^A^	*p* value
HVLT-R immediate recall	− 0.52	− 4.95	< 0.001*	− 0.52 (medium)	27.2	4.9	18.60	< 0.001*
HVLT-R delayed recall	− 0.27	− 2.59	0.010*	− 0.27 (small)	15.2	4.8	6.04	0.014*
HVLT-R recognition	− 0.21	− 1.99	0.047*	− 0.21 (small)	14.3	8.7	1.54	0.215
TMT A	− 0.99	− 9.21	< 0.001*	− 0.99 (large)	25.3	7.7	11.08	0.001*
TMT B|A^c^	− 1.49	− 13.35	< 0.001*	− 1.49 (large)	28.8	5.8	17.99	< 0.001*
COWA	− 0.63	− 6.06	< 0.001*	− 0.63 (medium)	27.2	7.7	13.23	< 0.001*
Digit span forward	− 0.43	− 4.10	< 0.001*	− 0.43 (small)	10.9	5.8	1.64	0.200
Digit span backward	− 0.78	− 7.51	< 0.001*	− 0.78 (medium)	22.8	6.8	10.15	0.001*
Digit symbol	− 1.49	− 13.78	< 0.001*	− 1.49 (large)	55.3	6.7	54.05	< 0.001*
GP dominant hand	− 1.43	− 13.42	< 0.001*	− 1.43 (large)	27.3	6.9	14.41	< 0.001*
GP non-dominant hand	− 1.63	− 15.25	< 0.001*	− 1.63 (large)	43.2	5.9	36.94	< 0.001*

*HVLT-R* Hopkins verbal learning test revised, *TMT* trail making test, *COWA* Controlled Oral Word Association, *GP* grooved pegboard

^*^p ≤ 0.05 (group-level) and *p* ≤ 0.04 (individual-level): alpha was corrected using the Benjamini–Hochberg method Benjamini and Hochberg [35]

^a^One-tailed one-sample z tests (*N controls* = 104; *M* = 0; *SD* = 1; *N patients* = 80–92)

^b^Cognitive impairment was defined as a z score ≤ − 1.5 (*N patients* = 80–92*; N controls* = 102–104)

^c^TMT B|A: Trails B score adjusted for sex, age, educational level and the Trails A score

^d^Higher z scores reflect better performance

^e^Glass’ delta: Interpretable as Cohen’s *d* effect sizes: ≥ 0.20–0.49 = small, ≥ 0.50–0.79 = medium, ≥ 0.9 = large [34]

^A^Chi-square test of homogeneity

### Individual cognitive performance

Percentages of impairment on all 11 test variables were higher in patients than in non-cancer controls. This difference was statistically significant, except for verbal recognition and attention (Table 3). These percentages were highest for information processing (55.3%), dexterity (43.2%; non-dominant hand) and cognitive flexibility (28.8%). Compared to controls, more patients showed cognitive impairments in more tests (Table 4). Significantly more patients (62% and 46%) than controls (18% and 3%) had an impairment in at least two or three test variables respectively.

**Table 4:** Cognitive performance at the individual level impairment on one or more test variables

No. of tests	Patients (%) (n = 76)	Controls (%) (n = 99)	*χ*^*2*^^b^	*p* value
≥ 1 test	76.3	43.4	19.05	< 0.001^c^
≥ 2 tests	61.8	18.2	35.10	< 0.001^c^
≥ 3 tests	46.1	3.0	46.81	< 0.001^c^
≥ 4 tests	36.8	3.0	33.72	< 0.001^c^
≥ 5 tests	23.7	0	26.14	< 0.001^c^
≥ 6 tests	14.5	0	15.29	< 0.001^c^
≥ 7 tests	11.8	0	12.36	< 0.001^c^
≥ 8 tests	6.6	0	6.71	0.010^c^
≥ 9 tests	0	0	N/A	N/A
≥ 10 tests	0	0	N/A	N/A
11 tests	0	0	N/A	N/A

^a^Impaired performance (z score ≤ -− 1.5) of patients with complete test scores on all tests. For 16 patients (17.4%) and 5 controls (4.8%) scores on one or more tests were missing due to: invalid assessment (HVLT-R recognition, TMT), unfamiliarity with the alphabet (TMT), visual problems (TMT, Digit Symbol, GP), and impairments in manual dexterity (TMT, Digit Symbol, GP)

^b^Chi-square test of homogeneity

^c^Statistical significance was considered as *p* ≤ 0.05: alpha was corrected according to the Benjamini–Hochberg method Benjamini and Hochberg [35]

### Predictors of baseline cognitive performance

Supplementary Tables 1 and 2 present the results of the exploratory correlation analyses (Online Resource 1). A metachronous diagnosis of BM (compared to synchronous) was significantly associated with worse performance on 7 out of the 11 test variables. Chemotherapy was significantly negatively correlated with performance on 3 test variables (immediate and delayed memory and psychomotor speed). Mental fatigue was significantly negatively associated with psychomotor speed, information processing, and dexterity. Higher KPS was significantly associated with greater dexterity.

Four additional clinical (KPS; chemotherapy; symptomatic versus asymptomatic BM; timing of BM diagnosis) and four psychological predictors (Reduced Activation; Reduced Motivation; Mental Fatigue; symptoms of depression) were selected for the hierarchical multiple regression analyses. None of the initial regression models with only number and volume of the BM as predictors, nor the predictors themselves, were statistically significant (Table 5). The addition of the clinical and psychological predictors led to a statistically significant increase in explained variance in five models for measures of verbal memory, psychomotor speed, information processing and dexterity. In two models (delayed recognition and information processing), timing of BM diagnosis was the only significant predictor, whereby patients with metachronous BM performed worse. Post hoc descriptive analyses showed that of the patients with a metachronous diagnosis, 44% had NSCLC, 55% received (prior) chemotherapy and 53% had a high KPS of 90–100 (vs. 96%, 7% and 89% in the synchronous group, respectively). For immediate verbal memory, symptomatic (versus asymptomatic) BM was a significant predictor, whereby patients with symptomatic BM performed worse. For psychomotor speed, mental fatigue was the only significant predictor in the model, with slower psychomotor speed in patients with more symptoms of mental fatigue. A final significant model did not yield any significant individual predictors (dexterity non-dominant hand).

**Table 5:** Multiple hierarchical regression predicting patients’ cognitive test performance

Test variable	Model	Predictor	B	*SE* B	p*	F(*df*)	*R*^2^	*∆R*2	p* (*∆R*^2^)
HVLT-R immediate recall	Model 1				0.220	1.50 (3,88)	0.049		
		Number of BM_Single_	− 0.635	0.363	0.084				
		Number of BM_5–10_	0.023	0.366	0.949				
		Total volume of BM	0.002	0.020	0.922				
	Model 2				0.011*	2.96 (6,85)	0.173	0.124	0.008*
		Number of BM_Single_	− 0.554	0.353	0.120				
		Number of BM_5–10_	− 0.081	0.352	0.818				
		Total volume of BM	0.014	0.020	0.474				
		Chemotherapy	− 0.402	0.319	0.211				
		Symptomatic (y/n)	− 0.743	0.318	0.022*				
		Timing of BM diagnosis	− 0.472	0.343	0.173				
HVLT-R delayed recall	Model 1				0.046	2.77 (3,88)	0.086		
		Number of BM_Single_	− 0.540	0.323	0.098				
		Number of BM_5–10_		0.326	0.754				
		Total volume of BM	0.103	0.017	0.102				
	Model 2				0.013*	3.10 (5,86)	0.153	0.066	0.039*
		Number of BM_Single_	− 0.388	0.320	0.229				
		Number of BM_5–10_	0.095	0.319	0.766				
		Total volume of BM	− 0.030	0.017	0.079				
		Chemotherapy	− 0.291	0.290	0.319				
		Timing of BM diagnosis	− 0.533	0.308	0.087				
HVLT-R recognition	Model 1				0.426	0.94 (3,87)	0.031		
		Number of BM_Single_	− 0.166	0.356	0.642				
		Number of BM_5–10_	0.071	0.359	0.844				
		Total volume of BM	− 0.028	0.019	0.149				
	Model 2				0.014*	3.04 (5,85)	0.151	0.120	0.004*
		Number of BM_Single_	− 0.049	0.345	0.888				
		Number of BM_5–10_	− 0.049	0.342	0.887				
		Total volume of BM	− 0.019	0.019	0.313				
		Symptomatic (y/n)	− 0.568	0.308	0.068				
		Timing of BM diagnosis	− 0.790	0.300	0.010*				
TMT A	Model 1				0.135	1.91 (3,82)	0.065		
		Number of BM_Single_	− 0.425	0.458	0.356				
		Number of BM_5–10_	0.541	0.459	0.242				
		Total volume of BM	− 0.025	0.025	0.302				
	Model 2				0.005*	3.42 (6,79)	0.206	0.141	0.005*
		Number of BM_Single_	− 0.172	0.438	0.695				
		Number of BM_5–10_	0.504	0.433	0.249				
		Total volume of BM	− 0.020	0.023	0.389				
		Chemotherapy	− 0.572	0.398	0.154				
		Timing of BM diagnosis	− 0.299	0.434	0.492				
		Mental fatigue	− 0.109	0.046	0.021*				
TMT B|A	Model 1				0.683	0.501 (3,76)	0.019		
		Number of BM_Single_	− 0.567	0.723	0.435				
		Number of BM_5-10_	− 0.466	0.709	0.513				
		Total volume of BM	− 0.028	0.038	0.455				
COWA	Model 1				0.289	1.27 (3,87)	0.042		
		Number of BM_Single_	− 0.515	0.315	0.106				
		Number of BM_5-10_	− 0.058	0.318	0.856				
		Total volume of BM	− 0.006	0.017	0.708				
	Model 2				0.091	1.97 (5,85)	0.104	0.062	0.059
		Number of BM_Single_	− 0.419	0.312	0.183				
		Number of BM_5–10_	− 0.036	0.311	0.908				
		Total volume of BM	− 0.010	0.017	0.562				
		Timing of BM diagnosis	− 0.360	0.275	0.195				
		Reduced motivation	− 0.059	0.033	0.078				
Digit span forward	Model 1				0.741	0.417 (3,88)	0.014		
		Number of BM_Single_	0.015	0.240	0.950				
		Number of BM_5–10_	0.069	0.242	0.777				
		Total volume of BM	− 0.014	0.013	0.276				
Digit span backward	Model 1				0.163	1.75 (3,88)	0.083		
		Number of BM_Single_	− 0.128	0.267	0.632				
		Number of BM_5–10_	− 0.144	0.269	0.594				
		Total volume of BM	− 0.029	0.014	0.046				
	Model 2				0.108	1.96 (4,87)	0.083	0.026	0.118
		Number of BM_Single_	− 0.167	0.266	0.532				
		Number of BM_5–10_	− 0.188	0.268	0.486				
		Total volume of BM	− 0.022	0.015	0.138				
		Symptomatic (y/n)	− 0.379	0.240	0.118				
Digit symbol	Model 1				0.518	0.764 (3,80)	0.028		
		Number of BM_Single_	0.063	0.343	0.855				
		Number of BM_5–10_	− 0.033	0.353	0.925				
		Total volume of BM	− 0.028	0.019	0.150				
	Model 2				0.010*	3.03 (6,77)	0.191	0.163	0.003*
		Number of BM_Single_	0.226	0.324	0.488				
		Number of BM_5–10_	− 0.058	0.328	0.859				
		Total volume of BM	− 0.023	0.018	0.210				
		Timing of BM diagnosis	− 0.625	0.295	0.037*				
		Mental fatigue	− 0.041	0.038	0.281				
		Symptoms of depression	− 0.068	0.037	0.072				
GP dominant hand	Model 1				0.511	0.78 (3,83)	0.027		
		Number of BM_Single_	− 0.720	0.771	0.353				
		Number of BM_5–10_	− 0.558	0.787	0.480				
		Total volume of BM	− 0.038	0.041	0.361				
	Model 2				0.194	1.56 (4,82)	0.070	0.043	0.054
		Number of BM_Single_	− 0.602	0.761	0.431				
		Number of BM_5–10_	− 0.555	0.774	0.475				
		Total volume of BM	− 0.030	0.041	0.466				
		Mental fatigue	− 0.152	0.078	0.054				
GP non-dominant hand	Model 1				0.977	0.07(3,84)	0.002		
		Number of BM_Single_	− 0.238	0.601	0.693				
		Number of BM_5–10_	− 0.238	0.609	0.697				
		Total volume of BM	− 0.002	0.032	0.961				
	Model 2				0.018*	2.73(6, 81)	0.168	0.166	0.002*
		Number of BM_Single_	− 0.137	0.576	0.813				
		Number of BM_5–10_	− 0.312	0.571	0.587				
		Total volume of BM	− 0.002	0.030	0.949				
		KPS	0.031	0.029	0.284				
		Timing of BM diagnosis	− 1.03	0.526	0.054				
		Reduced activity	− 0.117	0.062	0.062				

Bold values indicate the statistically significant difference

*HVLT-R* Hopkins verbal learning test revised, *TMT* trail making test, *COWA* Controlled Oral Word Association, *GP* grooved pegboard, *BM* brain metastases, *KPS* Karnofsky Performance Index, *B* unstandardized regression coefficient, *SE B* standard error B, *df* degrees of freedom

Coding of predictors: single BM: Number of BM_single_ = 1; 2–4 BM: Number of BM_single_ = 0, Number of BM_5-10_ = 0, 5–10 BM: Number of BM_5-10_ = 1; Symptomatic: yes = 1, no/asymptomatic = 0; Timing of BM diagnosis: synchronous = 0, metachronous = 1

*Statistical significance was considered as *p* ≤ 0.005 (models 1) and *p* ≤ 0.03 (models 2), alpha was corrected according to the Benjamini–Hochberg method [35] and as *p* ≤ 0.05 for the individual regression coefficients and change in R^2^ per model

## Discussion

In this study we examined the incidence and severity of cognitive impairment, and clinical as well as psychological predictors thereof, in selected patients with 1–10 BM who were accepted for GKRS. Cognitive performance was measured with a well-established neuropsychological test battery. Previous studies on cognitive functioning were focused on patients with 1–4 BM or made use of an insensitive measure to assess cognitive test performance (the MMSE) [5].

At group level, we found lowest cognitive test performance (large effect sizes; means that ranged between − 1 and − 1.6 SD below the normative mean) on measures of psychomotor speed, cognitive flexibility, information processing, and dexterity of both dominant and non-dominant hand. At the individual level, cognitive performance was most frequently impaired with respect to measures of short-term verbal memory span, cognitive flexibility, information processing, and dexterity of both dominant and non-dominant hand. Although at group level, patients performed significantly worse than controls (with small effect sizes) on measures of verbal recognition and immediate attention. At the individual level, however, there were no significant differences in the frequencies of impairment for these two measures. These results are largely in line with previous studies in patients with BM: cognitive impairment in one or more tests before treatment of BM ranged between 53 and 80% (76% in our sample) and was most clearly demonstrated in the domains of executive functioning (including cognitive flexibility), verbal and visual memory, dexterity and psychomotor speed [6, 7, 9, 10, 36, 37].

We noted a degree of impairment in information processing in our study that is higher than in other studies. Some of these studies used different neuropsychological tests, however, both studies by Chang et al. [6, 7] used the WAIS Digit Symbol test as well. At baseline, only 7% of their patients showed impaired performance in the pilot study [6] and baseline z scores in the larger randomized trial ranged between − 0.1 and − 0.4 [7] whereas in our sample, 55% of patients had impaired performance on this test and the mean z score was − 1.5. This difference might be explained by differences in the study samples: compared to our study, their sample consisted of patients with fewer (1–3) BM, higher median KPS and lower median total BM volume BM. In addition, although having severe problems with dexterity was one of the exclusion criteria in our study, impairments in dexterity were (highly) prevalent in our patient sample: 27% of patients showed impaired dominant hand dexterity (the mean z score for this measure was − 1.43 in our study vs.  − 1.30 in the SRS-arm of Chang et al. [7]). These impairments may have influenced performance on the other measures with high dominant hand motor demands [38] and help explain the poor performance on information processing, psychomotor speed, and cognitive flexibility. The use of (additional) neuropsychological tests with minimal motor requirements should be considered in future trials in this patient population, as the assessment of speed (information processing or psychomotor) is aimed at understanding cognitive rather than physical function [38].

Multivariable regression was used to examine whether number or volume of BM was predictive of pretreatment cognitive test performance. Neither number nor volume of BM were significant predictors in any of these initial models. Similarly, in previous studies based on univariate analyses, number of BM was not associated with cognitive performance. However, the same studies found negative associations uncorrected for multiple testing between total BM volume and measures of attention, verbal memory, information processing and executive functions [6, 8, 10, 15]. We also found a significant negative univariate association between volume of BM and working memory but in multivariable analyses volume of BM was not a significant predictor of working memory.

Hierarchical multivariable models including clinical as well as psychological variables were predictive of performance on six measures of verbal memory, psychomotor speed, information processing, and dexterity. Timing of BM diagnosis was a significant individual predictor in two out of five significant regression models: patients with a synchronous (versus metachronous) diagnosis of BM performed better on verbal recognition and had higher information processing (speed). This might be explained by the fact that these patients were still largely treatment-naïve and were in a better overall (higher KPS), and cognitive condition. Patients with a metachronous diagnosis of BM on the other hand, already received various types of systemic treatment, including chemotherapy, for their primary tumor, which may have contributed to the cognitive impairments [39, 40] already before the diagnosis of the BM. These (cancer-related) cognitive impairments primarily involve the domains of memory, attention, executive functioning, and processing speed [41].

Despite the fact that the patients in our study had significantly more symptoms of anxiety and depression than our controls we found no evidence for a direct effect of anxiety and depression on cognitive test performance in our prediction models. This is in line with a previous study in patients with BM and indicates that anxiety and depression may not be (primary) contributors to cognitive impairment in these patients [37]. Mental fatigue however was predictive of reduced psychomotor speed. Efforts should be continued to investigate specific patient- and tumor-specific factors that can predict cognitive test performance. Identification of these characteristics allow for more individually tailored care for patients. In addition, thorough assessment of cognitive impairment, and understanding of the predictors thereof, is crucial for the evaluation of cognitive changes after SRS [4].

This study has some limitations to be considered. Our patients had BM originating from various primary tumor histologies. Since prognosis, systemic treatment, and timing of BM may vary with type of primary cancer [42], this might have affected cognitive test performance. However, as most BM originate from lung cancer, lung cancer patients represent the majority of patients with BM, both in clinical practice and in clinical trials (including this study). In addition, we did not examine or take into account the location(s) of the BM. Further study is required to examine the impact of BM location (e.g., supratentorial, cerebellar, brainstem and ‘other’) on cognitive test performance as cognitive impairment is related to the site of tumor growth [43]. Although we did not find a direct effect of number and volume of BM on cognitive test performance in our relatively large sample of patients with 1–10 BM, it is of interest to investigate whether change (reduction or progression) in number and volume influences change in cognitive test performances after SRS. Li et al. [44] showed that greater volume reduction in total volume of BM was associated with a delay in cognitive decline after WBRT [44].

Significant associations between cognitive test performance and daily functional independence have been found in brain tumor patients [45]. This study used mostly the same neuropsychological tests as the current study. Strongest associations were found for executive functioning (TMT B), language comprehension (COWA) and verbal learning and memory (HVLT-R). Patients with BM in our study showed significant impairments in all of these tests. These impairments may cause serious difficulties in day-to-day activities (e.g., daily chores, preparing dinner or communicating with family and friends). For example, patients may experience difficulties with the ability to plan ahead (related to impaired cognitive flexibility), slowness of comprehension and processing of information (related to impaired processing speed), and difficulties in learning and remembering new information (related to functions of memory), and difficulties in performing adequate movements appropriate to a certain task (related to impairments in dexterity and executive functioning). In addition, these difficulties in everyday living may increase the caregiver burden [45].

Assessment of cognitive deficits is also crucial in understanding patients’ ability in weighing the risks (cognitive impairment, distant recurrences, neurotoxicity) and benefits (cognitive preservation, local control, distant control) in coming to a treatment decision (e.g., WBRT, SRS or best supportive care) [46]. A previous study indicated that over half of the patients with BM (prior to BM treatment) had a diminished ability to reason through medical treatment decisions [47], this was associated (same study sample) with worse verbal memory and information processing [48, 49]. In our sample, 55% (information processing), 27% (immediate verbal memory and verbal fluency) and 23% (working memory) of patients had impairments in these cognitive domains, emphasizing the relevance of pretreatment neuropsychological assessment. Patients at risk may need additional (written) information and guidance through the process of understanding treatment choices. Early detection of these cognitive impairments may facilitate cognitive intervention planning. Intervention (e.g., cognitive rehabilitation programs; [50] at an early stage may benefit the quality of survival in these patients, which is of particular interest for the growing number of (subgroups of) patients with longer expected survival.

## Electronic supplementary material

Below is the link to the electronic supplementary material.

Supplementary file1 (PDF 77 kb)

## References

[1] Loeffler JS (2019) Overview of the treatment of brain metastases. Wen PY, ed. UpToDate. Waltham, MA: UpToDate Inc.https://www.uptodate.com. Accessed June 14 2019

[2] Witgert ME, Meyers CA (2011). Neurocognitive and quality of life measures in patients with metastatic brain disease. Neurosurg Clin N Am.

[3] Li J, Bentzen SM, Li J, Renschler M, Mehta MP (2008). Relationship between neurocognitive function and quality of life after whole-brain radiotherapy in patients with brain metastasis. Int J Radiat Oncol Biol Phys.

[4] Wefel JS, Parsons MW, Gondi V, Brown PD (2018). Neurocognitive aspects of brain metastasis. Handb Clin Neurol.

[5] Schimmel WCM, Gehring K, Eekers DBP, Hanssens PEJ, Sitskoorn MM (2018). Cognitive effects of stereotactic radiosurgery in adult patients with brain metastases: a systematic review. Adv Radiat Oncol.

[6] Chang EL, Wefel JS, Maor MH, Hassenbusch SJ, Mahajan A, Lang FF (2007). A pilot study of neurocognitive function in patients with one to three new brain metastases initially treated with stereotactic radiosurgery alone. Neurosurgery.

[7] Chang EL, Wefel JS, Hess KR, Allen PK, Lang FF, Kornguth DG (2009). Neurocognition in patients with brain metastases treated with radiosurgery or radiosurgery plus whole-brain irradiation: a randomised controlled trial. Lancet Oncol.

[8] Onodera S, Aoyama H, Tha KK, Hashimoto N, Toyomaki A, Terae S (2014). The value of 4-month neurocognitive function as an endpoint in brain metastases trials. J Neurooncol.

[9] Brown PD, Jaeckle K, Ballman KV, Farace E, Cerhan JH, Anderson SK (2016). Effect of radiosurgery alone vs radiosurgery with whole brain radiation therapy on cognitive function in patients with 1 to 3 brain metastases. JAMA.

[10] Habets EJJ, Dirven L, Wiggenraad RG, Verbeek-de Kanter A, Lycklama A, Nijeholt GJ, Zwinkels H (2016). Neurocognitive functioning and health-related quality of life in patients treated with stereotactic radiotherapy for brain metastases: a prospective study. Neuro-oncology.

[11] Mehta MP, Shapiro WR, Glantz MJ, Patchell RA, Weitzner MA, Meyers CA (2002). Lead-in phase to randomized trial of motexafin gadolinium and whole-brain radiation for patients with brain metastases: centralized assessment of magnetic resonance imaging, neurocognitive, and neurologic end points. J Clin Oncol.

[12] Brown PD, Ballman KV, Cerhan JH, Anderson SK, Carrero XW, Whitton AC (2017). Postoperative stereotactic radiosurgery compared with whole brain radiotherapy for resected metastatic brain disease (NCCTG N107C/CEC·3): a multicentre, randomised, controlled, phase 3 trial. Lancet Oncol.

[13] Berger A, Strauss I, Moshe SB, Corn BW, Limon D, Shtraus N (2018). Neurocognitive evaluation of brain metastases patients treated with post-resection stereotactic radiosurgery: a prospective single arm clinical trial. J Neurooncol.

[14] Welzel G, Fleckenstein K, Schaefer J, Hermann B, Kraus-Tiefenbacher U, Mai SK (2008). Memory function before and after whole brain radiotherapy in patients with and without brain metastases. Int J Radiat Oncol Biol Phys.

[15] Meyers CA, Smith JA, Bezjak A, Mehta MP, Liebmann J, Illidge T (2004). Neurocognitive function and progression in patients with brain metastases treated with whole-brain radiation and motexafin gadolinium: results of a randomized phase III trial. J Clin Oncol.

[16] Hunter GK, Suh JH, Reuther AM, Vogelbaum MA, Barnett GH, Angelov L (2012). Treatment of five or more brain metastases with stereotactic radiosurgery. Int J Radiat Oncol Biol Phys.

[17] Limon D, McSherry F, Herndon J, Sampson J, Fecci P, Adamson J (2017). Single fraction stereotactic radiosurgery for multiple brain metastases. Adv Radiat Oncol.

[18] Lam T-C, Sahgal A, Chang EL, Lo SS (2014). Stereotactic radiosurgery for multiple brain metastases. Expert Rev Anticancer Ther.

[19] Baschnagel AM, Meyer KD, Chen PY, Krauss DJ, Olson RE, Pieper DR (2013). Tumor volume as a predictor of survival and local control in patients with brain metastases treated with Gamma Knife surgery. J Neurosurg.

[20] Emery A, Trifiletti DM, Romano KD, Patel N, Smolkin ME, Sheehan JP (2017). More than just the number of brain metastases: evaluating the impact of brain metastasis location and relative volume on overall survival after stereotactic radiosurgery. World Neurosurg.

[21] Kotecha R, Miller JA, Chao ST, Mohammadi AM, Murphy ES, Suh JH (2017). What drives patient outcomes in brain metastases: number, volume, or biology?. J Clin Oncol Am Soc Clin Oncol.

[22] Sharma M, Jia X, Ahluwalia M, Barnett GH, Vogelbaum MA, Chao ST (2017). Cumulative intracranial tumor volume and number of brain metastasis as predictors of developing new lesions after stereotactic radiosurgery for brain metastasis. World Neurosurg.

[23] Sperduto PW, Kased N, Roberge D, Xu Z, Shanley R, Luo X (2012). Summary report on the graded prognostic assessment: an accurate and facile diagnosis-specific tool to estimate survival for patients with brain metastases. J Clin Oncol.

[24] Pulenzas N, Khan L, Tsao M, Zhang L, Lechner B, Thavarajah N (2014). Fatigue scores in patients with brain metastases receiving whole brain radiotherapy. Support Care Cancer.

[25] Pendergrass JC, Targum SD, Harrison JE (2018). Cognitive impairment associated with cancer: a brief review. Innov Clin Neurosci.

[26] Thong MSY, Mols F, van de Poll-Franse LV, Sprangers MAG, van der Rijt CCD, Barsevick AM (2018). Identifying the subtypes of cancer-related fatigue: results from the population-based PROFILES registry. J Cancer Surviv.

[27] Verhaak E, Gehring K, Hanssens PEG, Sitskoorn MM (2019). Health-related quality of life of patients with brain metastases selected for stereotactic radiosurgery. J Neurooncol.

[28] Wefel JS, Vardy J, Ahles T, Schagen SB (2011). International Cognition and Cancer Task Force recommendations to harmonise studies of cognitive function in patients with cancer. Lancet Oncol.

[29] Zigmond AS, Snaith RP (1983). The hospital anxiety and depression scale. Acta Psychiatr Scand.

[30] Smets EM, Garssen B, Cull A, De Haes JC (1996). Application of the multidimensional fatigue inventory (MFI-20) in cancer patients receiving radiotherapy. Br J Cancer.

[31] Weitzner MA, Meyers CA, Gelke CK, Byrne KS, Cella DF, Levin VA (1996). The Functional Assessment of Cancer Therapy (FACT) scale. Development of a brain subscale and revalidation of the general version (FACT-G) in patients with primary brain tumors. Cancer.

[32] Oosterhuis HEM, van der Ark LA, Sijtsma K (2015). Sample size requirements for traditional and regression-based norms. Assessment.

[33] Glass GV, McGaw B, Smith ML (1981). Meta-analysis in social research.

[34] Cohen J (1988) Statistical power analysis for the behavioural sciences. Hillsdale.

[35] Benjamini Y, Hochberg Y, Sijtsma K (2002). Controlling the False Discovery rate: a practical and powerful approach to multiple testing. J R Stat Soc Ser B.

[36] van der Meer PB, Habets EJJ, Wiggenraad RG, Verbeek-de Kanter A, Lycklama A, Nijeholt GJ, Zwinkels H (2018). Individual changes in neurocognitive functioning and health-related quality of life in patients with brain oligometastases treated with stereotactic radiotherapy. J Neurooncol.

[37] Gerstenecker A, Nabors LB, Meneses K, Fiveash JB, Marson DC, Cutter G (2014). Cognition in patients with newly diagnosed brain metastasis: profiles and implications. J Neurooncol.

[38] Low E, Crewther SG, Ong B, Perre D, Wijeratne T (2017). Compromised motor dexterity confounds processing speed task outcomes in stroke patients. Front Neurol.

[39] Kesler SR, Blayney DW, Ong B, Perre D, Wijeratne T (2017). Neurotoxic effects of anthracycline- vs nonanthracycline-based chemotherapy on cognition in breast cancer survivors. JAMA Oncol.

[40] Schagen SB, Muller MJ, Boogerd W, Mellenbergh GJ, van Dam FSAM (2006). Change in cognitive function after chemotherapy: a prospective longitudinal study in breast cancer patients. J Natl Cancer Inst.

[41] Wefel JS, Kesler SR, Noll KR, Schagen SB (2015). Clinical characteristics, pathophysiology, and management of noncentral nervous system cancer-related cognitive impairment in adults. CA Cancer J Clin.

[42] Stelzer KJ (2013). Epidemiology and prognosis of brain metastases. Surg Neurol Int.

[43] Dwan TM, Ownsworth T, Chambers S, Walker DJ, Shum DHK (2015). Neuropsychological assessment of individuals with brain tumor: comparison of approaches used in the classification of impairment. Front Oncol.

[44] Li J, Bentzen SM, Renschler M, Mehta MP (2007). Regression after whole-brain radiation therapy for brain metastases correlates with survival and improved neurocognitive function. J Clin Oncol.

[45] Noll KR, Bradshaw ME, Weinberg JS, Wefel JS (2018). Neurocognitive functioning is associated with functional independence in newly diagnosed patients with temporal lobe glioma. Neurooncol Pract.

[46] Zeng KL, Raman S, Sahgal A, Soliman H, Tsao M, Wendzicki C (2017). Patient preference for stereotactic radiosurgery plus or minus whole brain radiotherapy for the treatment of brain metastases. Ann Palliat Med.

[47] Triebel KL, Gerstenecker A, Meneses K, Fiveash JB, Meyers CA, Cutter G (2015). Capacity of patients with brain metastases to make treatment decisions. Psychooncology.

[48] Gerstenecker A, Duff K, Meneses K, Fiveash JB, Nabors LB, Triebel KL (2015). Cognitive predictors of reasoning through treatment decisions in patients with newly diagnosed brain metastases. J Int Neuropsychol Soc.

[49] Gerstenecker A, Meneses K, Duff K, Fiveash JB, Marson DC, Triebel KL (2015). Cognitive predictors of understanding treatment decisions in patients with newly diagnosed brain metastasis. Cancer.

[50] Gehring K, Sitskoorn MM, Gundy CM, Sikkes SAM, Klein M, Postma TJ (2009). Cognitive rehabilitation in patients with gliomas: a randomized, controlled trial. J Clin Oncol.

