# Supplementary material for: Cognitive functioning and predictors thereof in patients with 1–10 brain metastases selected for stereotactic radiosurgery
Source: J Neurooncol. 2019 Sep 24;145(2):265–76. doi: 10.1007/s11060-019-03292-y (PMC6856035; doi:10.1007/s11060-019-03292-y)
Supplement: Supplementary file 1 — Supplementary file1 (PDF 77 kb) [file 11060_2019_3292_MOESM1_ESM.pdf]

Journal of Neuro-Oncology - Online Resource 1

Cognitive functioning and predictors thereof in patients with 1-10 brain metastases selected for stereotactic radiosurgery

Wietske C.M. Schimmel, MSc[1,2,3], Karin Gehring, PhD[2,3], Patrick E. J. Hanssens, MD[1,2], AND Margriet M. Sitskoorn, PhD[2,3]
[1] Elisabeth-TweeSteden Hospital - Gamma Knife Center, Tilburg, The Netherlands
[2] Elisabeth-TweeSteden Hospital - Department of Neurosurgery, Tilburg, The Netherlands
[3] Tilburg University, Department of Cognitive Neuropsychology, Tilburg, The Netherlands

Corresponding author: w.c.m.schimmel@tilburguniversity.edu

Supplementary Table 1. Correlations Between Clinical Characteristics and Patients' Cognitive Test Performance [a]

	Number of BM	Total volume of BM	KPS	DS-GPA	Systemic therapy (y/n)	Chemo-therapy [c]	Sympt / Asympt BM (0/1)	Synchr / Metachr dx of BM (0/1)	Epileptic seizures (y/n)	Time from primary cancer dx to enrollment
HVLT-R immediate recall	.096	-.011	.103	.024	-.128	**-.234***	**-.236***	**-.284****	-.155	-.015
HVLT-R delayed recall	.154	-.189	.067	-.033	-.089	**-.233***	-.187	**-.271****	-.084	-.032
HVLT-R recognition	.028	-.160	.068	-.073	-.132	-.123	**-.260***	**-.299****	-.052	-.089
TMT A	.180	-.099	.024	-.186	-.123	**-.301****	.067	**-.260***	-.026	-.190
TMT B|A [b]	-.087	-.102	.160	.111	.033	-.024	-.212	-.195	.141	.049
COWA	.121	-.055	.036	.000	-.090	-.112	-.096	**-.213***	-.063	-.014
Digit Span forward	-.028	-.114	-.005	-.050	-.046	-.062	-.137	-.159	-.080	-.026
Digit Span backward	-.064	**-.230***	.042	-.153	.054	-.059	**-.212***	-.145	-.185	.094
Digit Symbol	.006	-.161	.205	-.089	-.067	-.196	-.139	**-.274***	-.009	-.039
GP dominant hand	.036	-.127	.140	.052	.043	-.059	-.107	-.160	-.145	.110
GP non-dominant hand	.036	-.007	**.301****	.177	-.151	-.104	-.080	**-.293****	.074	.084

* $p \leq .05$; ** $p \leq .01$. *Note.* KPS = Karnofsky performance scale; BM = brain metastases; DS-GPA = diagnosis-specific graded prognostic assessment; (A)Sympt: (a)symptomatic; Synchr: synchronous; Metachr: metachronous; dx = diagnosis. [a] Higher z scores reflect better performance. [b] TMT B|A: Trails B adjusted for sex, age, educational level and Trails A. [c] Alone or in combination with other systemic therapies.

Supplementary Table 2. Correlations Between Psychological Measures [a] and Patients' Cognitive Test Performance [b]

	General Fatigue	Physical Fatigue	Reduced Activity	Reduced Motivation	Mental Fatigue	Symptoms of Anxiety	Symptoms of Depression
HVLT-R immediate recall	-.064	.005	-.005	-.091	-.025	.111	.022
HVLT-R delayed recall	-.083	.029	-.035	-.108	-.108	.104	.004
HVLT-R recognition	.131	.186	.163	.091	-.006	.131	.143
TMT A	-.047	-.034	-.046	-.093	**-.346****	-.093	-.180
TMT B|A [c]	-.009	.046	.107	.172	.035	.042	-.117
COWA	.001	.016	-.002	**-.213***	-.191	-.084	-.198
Digit Span forward	-.048	-.002	.079	.047	-.098	-.130	-.158
Digit Span backward	.005	.077	.000	.001	-.203	-.143	-.115
Digit Symbol	-.166	-.096	**-.222***	-.189	**-.288****	-.211	**-.318****
GP dominant hand	-.102	-.013	-.150	-.090	**-.227***	-.114	-.179
GP non-dominant hand	-.106	-.035	**-.290****	**-.235***	**-.266***	-.124	-.177

* $p \leq .05$; ** $p \leq .01$. [a] Raw subscale scores from the Multidimensional Fatigue Inventory (MFI) and the Hospital Anxiety and Depression Scale (HADS); higher scores indicate more symptoms. [b] Higher z scores reflect better performance. [c] TMT B|A: Trails B adjusted for sex, age, educational level and Trails A.
